# Successful pregnancy and live birth from a hypogonadotropic hypogonadism woman with low serum estradiol concentrations despite numerous oocyte maturations: a case report

**DOI:** 10.1186/s12884-017-1510-6

**Published:** 2017-09-20

**Authors:** Kaori Matsumoto, Kazuhiko Imakawa, Chuyu Hayashi

**Affiliations:** 1 Nihon University Itabashi Hospital, 30-1 Ooyaguchi-kamimachi, Itabashi-ku, Tokyo, 173-8610 Japan; 2 Animal Resource Science Center, Graduate School of Agricultural and Life Sciences, The University of Tokyo, 3145 Ago, Kasama, Ibaraki, 319-0206 Japan; 3 Department of Obstetrics and Gynecology, Nihon University School of Medicine, 30-1 Ooyaguchi-kamimachi, Itabashi-ku, Tokyo, 173-8610 Japan

**Keywords:** Low estradiol, High progesterone, Luteinizing hormone, Hypogonadotropic hypogonadism, Hypothalamic amenorrhea, Follicular fluids, Controlled ovarian stimulation, Assisted reproductive technology

## Abstract

**Background:**

The increase in serum estradiol ﻿(E_2_) concentrations during the follicular phase becomes the index of oocyte maturation in vivo. When ovarian stimulation is performed to hypogonadotropic hypogonadism (HH) patients with only follicle stimulating hormone (FSH), proper increase in serum E_2_ concentrations is not observed. Even if oocytes are obtained, which usually have low fertilization rate. In this report, we would like to present an unique case, in which under low E_2_ concentrations and without luteinizing hormone (LH) administration, numerous mature oocytes could be obtained and a healthy baby delivered.

**Case presentation:**

During controlled ovarian stimulation (COS) with only recombinant follicular stimulating hormone (rFSH) administrations, a 26-year-old Japanese woman with hypothalamic amenorrhea (i.e., hypogonadotropic hypogonadism) developed numerous follicles despite low serum E_2_, 701 pg/ml, and high progesterone (P_4_) concentrations, 2.11 ng/ml, on the day of induced ovulation. However, 33 cumulus-oocyte complexes (COCs) were successfully obtained; following the embryo culture, four early embryos and six blastocysts were cryopreserved. This patient received hormone replacement therapy (HRT), during which one of six cryopreserved blastocysts was thawed and transferred into the uterine lumen. The patient became pregnant from the first transfer, went through her pregnancy without any complications, and delivered a healthy male baby in the 39th week. Low E_2_ concentrations in follicular fluids (FFs) are suggestive that aromatase and/or 17β-hydroxysteroid dehydrogenase (17β-HSD) could be low.

**Conclusions:**

Serum E_2_ concentrations may not be the most important index for oocyte maturation during COS, and suggested that oocyte maturation was in progress even under low serum E_2_ and high P_4_ conditions. Even if serum E_2_ concentrations did not properly increase, numerous mature oocytes could be obtained, resulting in the birth of a healthy baby.

**Electronic supplementary material:**

The online version of this article (10.1186/s12884-017-1510-6) contains supplementary material, which is available to authorized users.

## Background

20–50 pg/ml serum estradiol (E_2_) concentrations are detected in the early follicular phase and menstruation, while concentrations over 200 pg/ml are detected in the late follicular phase and pre-ovulation [1]. The increase in serum E_2_ concentrations is indicative of follicular development and the important index in oocyte maturation. Hypogonadotropic hypogonadism (HH) patients exhibit low serum follicle stimulating hormone (FSH) and luteinizing hormone (LH) concentrations, resulting in negligible estrogen activity [2–4]. When a HH patient is treated with FSH alone during the controlled ovarian stimulation (COS), low serum E_2_ concentrations and the low fertilization rate are observed. However, human menopausal gonadotropin (HMG) containing FSH and LH activity is administered, serum E_2_ concentrations and fertilization rate are increased [2]. Administration of exogenous LH to HH patients is required to increase in serum E_2_ concentrations properly and to obtain adequate oocytes quality, resulting in delivery of healthy babies [2, 3].

Here, we report a case of a HH Japanese woman with daily administration of recombinant FSH (rFSH) alone, who had low serum E_2_ and high progesterone (P_4_) concentrations on the day of induced ovulation, but 33 cumulus-oocyte complexes (COCs) were collected. From these oocytes, good quality embryos were obtained and cryopreserved after in vitro fertilization (IVF) or intra cytoplasmic sperm injection (ICSI). A healthy male baby from one of the cryopreserved blastocysts was delivered.

## Case presentation

### Patient’s medical history

The patient, a 26-year-old Japanese woman, had been nulligravid. The first menstruation occurred at age 13 and had menstrual cycles regularly thereafter. At 21 years old, the patient experienced amenorrhea, resulting from 12 kg weight loss in two months. At another hospital, beginning at 23 years old, this patient had received oral E_2_ and P_4_ replacement therapy for two years. The patient was married at 26 years old and decided to undergo fertility treatment at our hospital. The patient’s body mass index (BMI) was 19.2 kg/m^2^ and her feminine characteristics appeared normal. The diameter of right ovary was 26.0 mm and left ovary was 29.1 mm. Serum FSH and LH concentrations were 3.2 mIU/ml and 0.5 mIU/ml, respectively. Her endocrine test results were within reference ranges of our hospital except for triiodothyronine (T_3_) and prolactin (PRL) (Additional file 1).The patient was diagnosed with hypothalamic amenorrhea following luteinizing hormone-releasing hormone (LH-RH) loading test.

Both oviducts and the uterine lumen in this patient were morphologically normal when examined with hysterosalpingography (HSG). The husband’s semen quality appeared normal and fell within the World Health Organization criteria 2010 [5]. Through the examination by transvaginal ultrasonography, over five antral follicles (i.e., 5–10 mm) each were observed in both ovaries. Serum Anti-Müllerian Hormone (AMH) concentrations were 10.8 ng/ml, from which the ovarian reserve was judged to be functioning [6–9]. To undergo planned infertility treatment for this couple, who lived in separately, the husband’s sperm were cryopreserved.

In our country, however, recombinant LH (rLH) is not yet available, and HMG and human chorionic gonadotropin (hCG) must be injected intramuscularly only by a medical doctor or nurse. Due to her work assignment and work loads, she was not able to receive daily administrations of HMG or hCG, therefore, self-administration of rFSH was only what we could offer for this patient during the COS period. Antral follicle counts (AFCs) and AMH concentrations of the patient were at high risk of ovarian hyperstimulation syndrome (OHSS) [10, 11]. Therefore, gonadotropin releasing hormone (GnRH) antagonist regimen was planned to avoid rapidly rising serum E_2_ concentrations [12, 13], only if proper increase in serum E_2_ concentrations were observed and dominant follicles reached over 12–14 mm in diameter.

### First ovarian stimulation

**Fig. 1:**
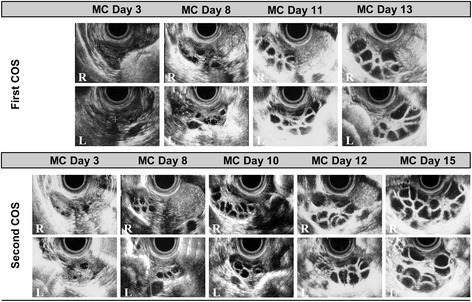
Follicular development following controlled ovarian stimulation (COS). Ovarian response was monitored through the use of transvaginal ultrasonography during COS. During the first COS, measurable follicles were not observed in either ovary on day 3 of the menstrual cycle (MC). On the same day, COS was initiated with daily administration of 225 international unit (IU) recombinant follicle stimulating hormone (rFSH). Follicular development and increased progesterone (P_4_) concentrations were observed, but estradiol (E_2_) concentrations were low on day 11. Although the right (R) and left (L) ovaries had over 30 follicles of 15–19 mm on day 13, serum E_2_ concentrations were low at 484 pg/ml and P_4_ concentrations were high at 2.09 ng/ml. COS was canceled due to the hormone concentration which did not reflect those of numerous follicular developments. From the day when the first COS was canceled, the patient took 10 mg synthetic progesterone (SP) orally for the next 14 days. The patient experienced withdrawal bleeding, and several small follicles were observed in both ovaries on day 3. From the same day, the daily dosage of 175 IU rFSH was administered for the first 5 days, followed by 200 IU for the remaining treatment period. Similar to the response seen at the first COS, high serum P_4_ concentrations, 2.11 ng/ml, were observed; however, serum E_2_ concentrations, 701 pg/ml, did not reflect those of numerous follicular developments

When this patient experienced withdrawal bleeding following daily administration of oral contraceptive (OC) for 14 days, measurable follicles were not observed in either ovary on day 3 (Fig. 1). On the same day, COS was initiated with daily administration of 225 IU rFSH. Follicular developments and increased P_4_ concentrations were observed, but E_2_ concentrations were low on day 11 (Table 1). GnRH antagonist regimen was not performed because proper increase in serum E_2_ concentrations was not observed. Although both ovaries had over 30 follicles of 15–19 mm on day 13, serum E_2_ concentrations were low at 484 pg/ml and P_4_ concentrations were high at 2.09 ng/ml. COS was canceled due to the hormone concentration which did not reflect those of numerous follicular developments.

**Table 1:** Transition in serum steroid hormone concentrations and follicular development during controlled ovarian stimulation

First COS					
MC day	MC Day 3	MC Day 8	MC Day 11	MC Day 13	
(rFSH administration)	(0 IU)	(1125 IU)	(1800 IU)	(2250 IU)	
E_2_	10^a^	–	219	484	
(pg/ml)					
LH	0.1^a^	–	0.1^a^	0.1^a^	
(mIU/ml)					
FSH	0.1	–	–	–	
(mIU/ml)					
P_4_	–	–	1.05	2.09	
(ng/ml)					
Follicular diameter (mm)	NM	6–8	9–12	15–19	
Second COS					
MC day	MC Day 3	MC Day 8	MC Day 10	MC Day 12	MC Day 15
(rFSH administration)	(0 IU)	(875 IU)	(1225 IU)	(1625 IU)	(2225 IU)
E_2_	10^a^	–	60	201	701
(pg/ml)					
LH	0.1^a^	–	0.1^a^	0.1^a^	0.1^a^
(mIU/ml)					
FSH	0.2	–	–	–	–
(mIU/ml)					
P_4_	**–**	–	0.40	0.53	2.11
(ng/ml)					
Follicular diameter (mm)	6–8	6–8	10	13–15	14–22

*Note*: *COS* controlled ovarian stimulation, *MC* menstrual cycle, *IU* international units, *NM* not measurable, *bar* no data, *E*_*2*_ estradiol, *LH* luteinizing hormone, *FSH* follicle stimulating hormone, *P*_*4*_ progesterone

^a^under detection limit

### Second controlled ovarian stimulation

From the day when the first COS was canceled, the patient took 10 mg synthetic progesterone (SP) orally for the next 14 days. The patient experienced withdrawal bleeding, and several small follicles were observed in both ovaries on day 3 (Fig. 1). From the same day, the daily dosage of 175 IU rFSH was administered for the first 5 days, followed by 200 IU for the remaining treatment period. Similar to the response seen at the first COS, high serum P_4_ concentrations were observed; however, serum E_2_ concentrations were not increased on day 15 (Table 1).

During COS, therefore, we advised this patient that assisted reproductive technology (ART) might fail due to her unusual serum steroid hormone concentrations, low E_2_ and high P_4_. At the same time, we also informed the patient that ART might succeed because serum AMH concentrations were adequate and numerous follicles were developed in response to rFSH. The patient and husband voluntarily agreed to go through ART in understanding the procedure with informed consent. On the same day, the ovulation was induced with 5000 IU hCG.

### IVF/ICSI, embryo culture and cryopreservation

35 h after the hCG administration, follicles were pricked with an oocyte pick-up (OPU) needle transvaginally. Follicular Fluids (FFs) from the largest follicle were aspirated, which were frozen for the subsequent hormone analysis. Sperm that had been cryopreserved were thawed, which had characteristics of 44.0 × 10^6^/ml and 9.5% motility. It was previously reported that oocytes from low serum E_2_ concentrations of a HH patient have decreased fertilization rate with IVF [2]. To obtain as many fertilized oocytes with sufficient quality frozen-thawed sperm, in addition to IVF, ICSI was also performed. 18 and 15 COCs were subjected to IVF and ICSI, respectively. IVF-COCs were inseminated with 5.0 × 10^4^ motile sperm/COC. After 16–18 h after IVF and ICSI, fertilization was determined by the presence of two pronuclei (PN). Fertilized oocytes were cultured in the cleavage medium for the initial 3 days, and embryos were then cultured in the blastocyst medium until blastocyst formation. Embryo cryopreservation was performed with an ultra-rapid vitrification kit.

### Hormone treatment, frozen-thawed blastocyst transfer and clinical pregnancy

Frozen-thawed blastocyst transfer (FBT) was performed following hormone replacement treatment (HRT) due to hypothalamic amenorrhea [14, 15]. After the patient experienced withdrawal bleeding by daily administration of OC for 14 days, daily administration of oral 2.0 mg E_2_ was initiated on day 3. Dosage of E_2_ was increased to 3.0 mg/day on day 6 and 4.0 mg/day on day 8. On day 11, serum E_2_ concentrations reached to 215 pg/ml and endometrial thickness was 12.0 mm, both of which exceeded our hospital standards of over 200 pg/ml serum E_2_ concentrations and endometrial thickness over 8.0 mm. The ovulation day (Ovd) was estimated to be on day 12 of the menstrual cycle. From the next day, luteal function was supported by daily administration of 30 mg oral SP, followed by 125 mg i.m. SP in every 5 days, which was continued until eight weeks of pregnancy.

A cryopreserved blastocyst was thawed with a thawing kit. FBT was performed transvaginally using an embryo transfer (ET) catheter under transabdominal ultrasound guidance. Serum hCG concentrations were determined 7 days after FBT and clinical pregnancy was confirmed with a transvaginal ultrasonography.

## Results of ART and Pregnancy

### IVF/ICSI and frozen-thawed blastocyst transfer

Among 33 COCs collected, 2PN rates were 77.8% (14/18) from IVF and 73.3% (11/15) from ICSI. Following the embryo culture, two early embryos (i.e., 7–10 blastomeres, culture on day 3) and five blastocysts were found from the IVF, and two early embryos and one blastocyst from the ICSI, all of which were cryopreserved. At day 5 from Ovd at the HRT cycle, one frozen-thawed blastocyst from the IVF, classified 5AA according to Gardner’s method [16], was transvaginally transferred into the uterine lumen.

### Pregnancy outcome and hormone concentrations throughout pregnancy period

Serum hCG concentrations, 87.4 mIU/ml, were detected on day 7 following the FBT procedure. The presence of a germinal sac was confirmed on the third day of the fifth week (5w3d) and fetal heartbeats were detected on the first day of the seventh week (7w1d) during her pregnancy period. In this patient, serum steroid hormone concentrations transitioned as expected (Table 2), and went through her pregnancy without any complications. The patient gave birth to a baby boy via vaginal delivery at the beginning of the 39th week (39w0d). The male baby without anomalies was weighed at 3288 g and scaled 9 points on Apgar Score.

**Table 2:** Hormone concentrations throughout her pregnancy period

	estradiol (pg/ml)	androstenedione (ng/ml)	testosterone (ng/ml)	DHEA-S (μg/dl)
15w2d	8,410	7.2	0.95	111
19w6d	14,500	8.5	1.09	114
30w2d	37,300	12.0	1.26	105

Note: *w* week, *d* day, *DHEA-S* dehydroepiandrosterone sulfate

### Steroid hormone concentrations and its hormonal precursor/hormone ratios in follicular fluids

Steroid hormone concentrations in this patient’s FFs were compared to those of infertile patients (Inf-) with similar serum E_2_ concentrations. These women, Inf-A and Inf-B, whose informed consents had been obtained and OPU performed in our hospital were both 700 ± 50 pg/ml serum E_2_ concentrations on the day of induced ovulation.

On day 4 of the menstrual cycle, these infertile women received daily administration of 100 mg clomifene citrate (CC) for 5 days. Follicular diameters were 25.2 mm and 18.1 mm for Inf-A and 22.8 mm for Inf-B on the day of induced ovulation. From the same day, serum hormone concentrations for Inf-A and Inf-B were 692 pg/ml and 745 pg/ml E_2_, 10.5 mIU/ml and 8.0 mIU/ml LH, and 0.40 ng/ml and 0.29 ng/ml P_4_, respectively. Ovulation was induced with the administration of 600 μg GnRH agonist, followed by the same procedures of OPU, IVF and FFs cryopreservation. However, no oocyte was collected from Inf-A, while one oocyte was collected from Inf-B, but its development ceased after fertilization.

**Fig. 2:**
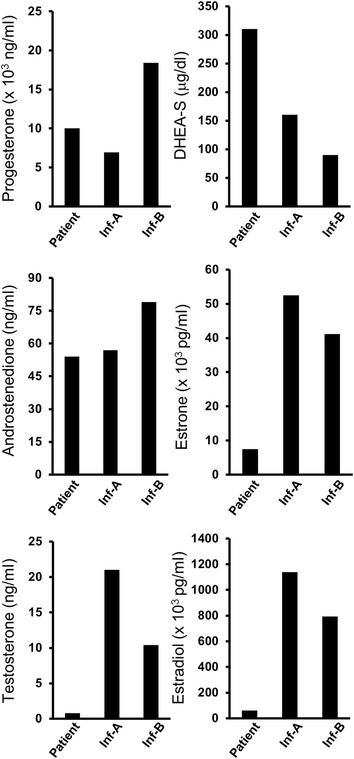
Comparison of steroid hormone concentrations of this patient to those of infertile patients (Inf-), Inf-A and Inf-B. On day 4 of the menstrual cycle, these infertile women received daily administration of 100 mg clomifene citrate (CC) for 5 days. Follicular diameters were 25.2 mm and 18.1 mm for Inf-A and 22.8 mm for Inf-B on the day of induced ovulation. From the same day, serum hormone concentrations for Inf-A and Inf-B were 692 pg/ml and 745 pg/ml E_2_, 10.5 mIU/ml and 8.0 mIU/ml LH, and 0.40 ng/ml and 0.29 ng/ml P_4_, respectively. Ovulation was induced with 600 μg gonadotropin releasing hormone (GnRH) agonist, followed by oocyte pick-up (OPU), in vitro fertilization (IVF) and follicular fluids (FFs) cryopreservation in the same manner. However, no oocyte was collected from Inf-A, while one oocyte was collected from Inf-B, but its development ceased after fertilization. Although progesterone (P_4_), dehydroepiandrosterone sulfate (DHEA-S) and androstenedione (A_2_) concentrations in FFs of this patient did not differ from those of Inf-A and Inf-B, estrone (E_1_), testosterone (T) and estradiol (E_2_) concentrations were lower than those of Inf-A and Inf-B

In this patient, P_4_, dehydroepiandrosterone sulfate (DHEA-S) and androstenedione (A_2_) concentrations in FFs did not differ from those of Inf-A and Inf-B, but estrone (E_1_), testosterone (T) and E_2_ concentrations were lower than those of Inf-A and Inf-B (Fig. 2). Although the T/E_2_ ratio did not differ, the A_2_/E_1_, A_2_/T and E_1_/E_2_ ratios of the patient were lower than those of the infertile women (Table 3).

**Table 3:** Hormonal Precursor/Hormone Ratios

	A_2_/E_1_	T/E_2_	A_2_/T	E_1_/E_2_
Enzyme involved	Aromatase	Aromatase	17β-HSD	17β-HSD
Patient	138.6	76,875.0	0.015	8.2
Inf-A	920.7	54,285.7	0.368	21.7
Inf-B	521.1	76,346.2	0.132	19.3

*Note*: Under bar denotes significantly lower in ratio than that of Inf-A and Inf-B

*E*_*1*_ estrone, *E*_*2*_ estradiol, *A*_*2*_ androstenedione, *T* testosterone, *17β-HSD* 17β-hydroxysteroid dehydrogenase

## Discussion

Here we report that a woman with hypothalamic amenorrhea became pregnant and delivered a healthy baby boy. During the follicular phase, the patient had low serum E_2_ and high P_4_ concentrations, followed by 33 COCs collection, to which IVF and ICSI were performed, and good early embryos and blastocysts were obtained. Although we carefully monitored this patient, her serum E_2_ concentrations quickly decreased and the ovaries were returned to the normal size 17 days after the first COS cancellation. She had similar condition at the time of the second COS cancellation, but symptoms of OHSS such abdominal pain and bloating, nausea, diarrhea and sudden weight increase were not observed. Observations in which concentrations of E_1_, T and E_2_ in FFs of this patient were found to be lower than those of the infertile women. When the ratios of steroid hormone concentrations of the patient were compared with those of the infertile women, the A_2_/E_1_, A_2_/T and E_1_/E_2_ ratios of the patient were lower than those of the infertile women. It appears that E_2_ was not sufficiently synthesized from A_2_. Low E_2_ concentrations in FFs suggest that aromatase and/or 17β-hydroxysteroid dehydrogenase (17β-HSD) levels and/or activities were low. It was possible that her hormone profile during COS could have been due to hereditary, her hormone profile was monitored throughout to the course of pregnancy, and that her hormone concentrations proceeded as healthy pregnancy. Both the patient and the child are healthy, and she delivered a female baby as second child through the use of a cryopreserved blastocyst obtained from the ART previously performed.

The increase in serum E_2_ concentrations during the follicular phase becomes the index of oocyte maturation in vivo; however, oocyte development in ovary undergoes even under E_2_ synthesis inhibition. It was reported that 7–16 mature oocytes were obtained under 500 pg/ml serum E_2_ concentrations on the day of induced ovulation, when COS with aromatase inhibitor was performed to women with breast cancer [17–19], and that number of mature oocytes and fertilization rates were not inferior to spontaneous COS [17, 19]. It has been noted that addition of E_2_ to the in vitro maturation (IVM) medium is not required [20, 21], suggesting that molecules other than E_2_ could be more important than steroid conditions [21–23].

Because serum P_4_ concentrations of the patient were higher than those in the infertile women, accumulation of P_4_ during COS was suspected. However, P_4_ concentrations in FFs did not differ between these patients, suggesting that P_4_ was not accumulated. In COS, infertile patients with numerous developing follicles tend to have increased serum P_4_ concentrations over 1.0 ng/ml. In this patient, increase in serum E_2_ concentrations did not reflect those of numerous follicular developments, possibly due to low aromatase and/or 17β-HSD, while serum P_4_ concentrations were sufficiently increased.

This case revealed that even if serum E_2_ concentrations were lower than those in the common practice, mature oocytes could be obtained and a healthy baby delivered. In the recent report, it was shown that mature oocytes are obtained even if COS was initiated in various phase of women’s menstrual cycle [24]. Together, these results suggest that E_2_ synthesis during COS and oocyte maturation could be more flexible than is commonly accepted.

## Conclusions

Serum E_2_ concentrations may not be the most important index for oocyte maturation during COS, and suggested that oocyte maturation was in progress even under low serum E_2_ and high P_4_ conditions. Even if serum E_2_ concentrations did not properly increase, numerous mature oocytes could be obtained, resulting in the birth of a healthy baby.

## Additional file

Additional file 1: Endocrine tests of the patient for infertility screening. (XLSX 9 kb)

## Abbreviations

17β-HSD: 17β-hydroxysteroid dehydrogenase
A_2_: Androstenedione
AFCs: Antral follicle counts
AMH: Anti-Müllerian Hormone
ART: Assisted reproductive technology
BMI: Body mass index
CC: Clomifene citrate
COCs: Cumulus-oocyte complexes
COS: Controlled ovarian stimulation
DHEA-S: Dehydroepiandrosterone sulfate
E_1_: Estrone
E_2_: Estradiol
ET: Embryo transfer
FBT: Frozen-thawed blastocyst transfer
FFs: Follicular Fluids
FSH: Follicle stimulating hormone
GnRH: Gonadotropin releasing hormone
hCG: Human chorionic gonadotropin
HH: Hypogonadotropic hypogonadism
HMG: Human menopausal gonadotropin
HRT: Hormone replacement treatment
HSG: Hysterosalpingography
ICSI: Intra cytoplasmic sperm injection
Inf-: Infertile patients
IVF: In vitro fertilization
IVM: In vitro maturation
LH: Luteinizing hormone
LH-RH: Luteinizing hormone-releasing hormone
OC: Oral contraceptive
OHSS: Ovarian hyperstimulation syndrome
OPU: Oocyte pick-up
Ovd: Ovulation day
P_4_: Progesterone
PN: Pronuclei
PRL: Prolactin
rFSH: Recombinant FSH
rLH: Recombinant LH
SP: Synthetic progesterone
T: Testosterone
T_3_: Triiodothyronine
